# Perspective of elderly patients on chronic use of potentially inappropriate medication – Results of the qualitative CIM-TRIAD study

**DOI:** 10.1371/journal.pone.0202068

**Published:** 2018-09-19

**Authors:** Kathrin Heser, Nadine Janis Pohontsch, Martin Scherer, Antje Löffler, Tobias Luck, Steffi G. Riedel-Heller, Wolfgang Maier, Debora Parker, Britta Haenisch, Frank Jessen

**Affiliations:** 1 Department for Neurodegenerative Diseases and Geriatric Psychiatry, University of Bonn, Bonn, Germany; 2 Department of General Practice / Primary Care, University Medical Center Hamburg-Eppendorf, Hamburg, Germany; 3 Institute of Social Medicine, Occupational Health and Public Health (ISAP), University of Leipzig, Leipzig, Germany; 4 Institute of Health Science, Brandenburg University of Technology (BTU) Cottbus-Senftenberg, Senftenberg, Germany; 5 Department of Economic and Social Sciences & Institute of Social Medicine, Rehabilitation Sciences and Healthcare Research (ISRV), University of Applied Sciences Nordhausen, Nordhausen, Germany; 6 German Center for Neurodegenerative Diseases (DZNE), Bonn, Germany; 7 Federal Institute for Drugs and Medical Devices (BfArM), Bonn, Germany; 8 Center for Translational Medicine, University of Bonn, Bonn, Germany; 9 Department of Psychiatry and Psychotherapy, University of Cologne, Cologne, Germany; University of Brescia, ITALY

## Abstract

Although potentially inappropriate medication (PIM) is associated with risk of harm due to adverse effects, it is frequently prescribed for elderly patients. The aim of this qualitative multi-center study was to gain insight into contextual factors that might lead to chronic PIM use. We conducted semi-structured interviews with elderly patients with or without chronic PIM use (patient interviews: n = 52). Patients were between 86 and 96 years old. The participants were recruited from the AgeCoDe study. Interviews were audiotaped and transcribed verbatim. The transcripts of the interviews were analysed using qualitative content analysis. Deductive and inductive categories were determined. We found contextual factors related to the patient and related to patient-general practitioner (GP) communication that might lead to chronic PIM use (i.e., positive features of PIM, maintaining characteristics of medication intake, barriers to deprescribe PIM, external actors supporting PIM intake, system-related factors). Besides certain health-related behaviours (e.g., own obligation to report to GP) and medication-related attitudes and knowledge (e.g., awareness of side effects and interaction of medicines), patient-GP-interactions that were characterised by mutual agreements on drugs (e.g., concerning dosage or discontinuation of a drug) might be advantageous to reduce the probability of chronic PIM use. The results might assist in the development of guidelines and educational programs aiming to reduce PIM use in the elderly.

## Introduction

Potentially inappropriate medication (PIM), which is associated with risk of harm due to side effects, is frequently prescribed for elderly patients (e.g., [1, 2]). The PRISCUS list summarizes individual drugs considered to be PIM in Germany [3]. Described concerns associated with these PIM drugs include, for example, gastrointestinal side effects, central nervous disturbances, falls, cardiac side effects, and cognitive impairment [3]. One quarter of German individuals of at least 65 years of age received at least one PRISCUS drug prescription within one year [4]. The intake of PIM was associated with an increased risk for hospitalization and death in nursing home residents of 65 years and older [5]. Chronic use of anticholinergic drugs, of which at least some have to be considered as PIM, was associated with an increased risk for dementia [6]. Other specified peripheral and central anticholinergic side effects of PIM drugs are constipation, dry mouth, orthostatic hypotension, cardiac arrythmia, drowsiness, inner unrest, confusion, and delirium [3].

Misunderstandings often occur in the general process of prescribing and are caused both by general practitioner (GP) and patient, but the role of patients´ participation needs to be emphasised particularly in the consultation [7]. Elderly patients felt comfortable with information about their medication when they trusted and felt confident about it, when they were satisfied with the information about the medication provided by the physician or self-acquired, and when they were able to take control during the information process [8]. Elderly multimorbid patients can understand the concept of competing outcomes of medication due to their experience of adverse drug effects, but the identification and prioritisation of global cross-disease health outcomes instead of disease-specific goals might improve patients´ complex health-care decisions according to Fried et al. [9]. Another qualitative study found that “knowledge (about disease and treatment) combined with faith in the doctor produces the motivation to start using medicines”([10] p. 369). A focus group study showed that elderly patients with multiple medicines both perceived the benefit and the risk of their drugs and valued the patient-physician relationship (in terms of trust/distrust and access) particularly regarding their medication [11]. Spinewine et al. [12] conducted a multi-method qualitative study and identified “reliance on general acute care and short term treatment”, “passive attitude towards learning”, and “paternalistic decision making” as categories that lead to an inappropriate use of medicines in elderly inpatients. Characteristics of shared-decision making were infrequently observed during medicine communications between patient and doctor, according to a study by Stevenson et al. [13].

The aim of the present study was to investigate contextual factors that lead to chronic PIM use in the elderly. Currently, there is a lack of qualitative studies on the perspective of the patient regarding the chronic prescription and intake of PIM, which leads to an incomplete understanding of the context in which it occurs. The patient interviews covered aspects of knowledge, health-related goals and values, and GP-patient interactions. Motivational factors for PIM prescription and potential barriers to PIM cessation were also addressed. Additionally, patients were asked about the effect of their medication on their health-related quality of life. These research questions are innovative and have not been targeted by qualitative interview studies so far to the best of our knowledge. Therefore, we provide additional insight into contextual factors from the elderly patient perspective on medication (including risks and benefits).

## Materials and methods

### Study design

The participants were recruited from the AgeCoDe study, which is a multi-center longitudinal study, initially with a total of 3327 patients of at least 75 years of age in 2003/2004, that were recruited from GP patient registries in Germany with frequent follow-up periods (e.g., [14]). Medication was documented at every AgeCoDe follow-up assessment. The present CIM-TRIAD study was conducted at three German study sites (Bonn, Hamburg, and Leipzig).

Semi-structured interviews were chosen as a qualitative method to obtain comprehensive explanations for context and individual factors that contribute to chronically inappropriate medication. Dyads or triads of elderly patients, their attending GPs, and relevant others were interviewed. The patient perspective was examined in the present study. Patients were not explicitly informed about PIM characteristics of their drugs in advance.

Cases of chronic PIM intake and matched cases without PIM (non-PIM) were identified by BH (pharmacist and pharmacologist) and DP of the group pharmacoepidemiology (led by BH). Criteria for PIM cases included an age of 75 years or older and the intake of at least one drug from the PRISCUS list [3] in the last follow-up intervals available. If available, preference was given to patients who were taking drugs from the PRISCUS list continuously for as many follow-up intervals as possible, including baseline. A non-PIM group was interviewed as well, in order to identify contextual factors that might decrease the probability of PIM prescription in very elderly patients. Controls did not take any drug from the PRISCUS list in baseline and any follow-up intervals. Controls were matched to cases on age (if available, ± one year, otherwise ± maximum five years), gender and the number of prescription drugs during the AgeCoDe study (baseline and all follow-up intervals available). For the number of prescribed drugs, the control with the number closest to the number of prescribed drugs of the case was chosen.

Identified cases were contacted and asked to participate in the study after the procedure of the study was explained. If patients consented to participate in the study, they were also asked for their permission to contact their GP and another significant person, to ask them also for their study participation. We aimed to conduct interviews of full triads containing all three perspectives. We obtained 52 patient interviews (PIM: n = 27, non-PIM: n = 25), 52 GP interviews (PIM: n = 25, non-PIM: n = 22, others: 5; for results see [15]), and 48 interviews of significant others of the patients (PIM: n = 24, non-PIM: n = 24). Interviews of the triad were usually conducted separately. Characteristics of the patients with and without chronic use of PIM are given in Tables 1 and 2.

**Table 1:** Characteristics of the patients with chronic use of potentially inappropriate medication (PIM).

Pseudonym	Gender	PIM drug
P1	female	Bromazepam, Flurazepam, Doxylamin
P2	female	Piracetam, Zopiclon*
P3	female	Bromazepam
P4	male	Bromazepam, Lorazepam*, Zopiclon*
P5	female	Acetyldigoxin
P6	female	Sotalol
P7	female	Trimipramin
P8	female	Sotalol, Acetyldigoxin, Amitriptylin
P9	male	Indometacin
P10	male	Sotalol
P11	female	Bromazepam, Doxylamin
P12	female	Bromazepam
P13	female	Lorazepam*, Trimipramin
P14	female	Nitrofurantoin
P15	male	Doxazosin (and Terazosin but due to its indication not a PIM)
P16	female	Nifedipin
P17	female	Dimenhydrinat in the past, not currently
P18	female	Solifenacin
P19	female	Piracetam
P20	female	Nitrazepam
P21	male	Piracetam
P22	female	Doxylamin
P23	female	Nitrazepam, Zopiclon*, Doxylamin
P24	female	Indometacin
P25	female	Piracetam
P26	female	Amitriptylin
P27	female	Piroxicam (formerly), Nicergolin (currently)

*Whether these drugs are considered as PIM depends on their dosage (definition according to the PRISCUS list [3]): Zopiclon > 3.75 mg/d, Lorazepam > 2 mg/d. All cases took at least one PIM; if dosage-dependent PIM were taken below the defined threshold, at least one other PIM above the threshold or without dosage definition was taken.

**Table 2:** Characteristics of the patients without chronic use of potentially inappropriate medication (non-PIM).

Pseudonym	Gender	PIM drug
C1	female	none
C2	female	none
C3	female	none
C4	male	none (Zopiclon only temporarily below PIM dose)
C5	female	none
C6	female	none
C7	female	none
C8	female	none
C9	male	none
C10	female	none
C11	female	none
C12	female	none
C13	male	none
C14	female	none
C15	female	none
C16	female	none
C17	female	none
C18	female	none
C19	female	none
C20	male	none
C21	female	none
C22	female	none
C23	female	none
C24	female	none
C25	female	none

### Development of the interview guideline

The interview guideline for the elderly patients was developed in Bonn (by KH and FJ). During the development of the interview guidelines, interdisciplinary exchange was obtained by the means of audioconferences and personal meetings. Audioconferences and personal meetings also served to assure homogenous implementation of the interview guideline and of the analysis strategies. Topics of the interviews were knowledge, awareness, risk monitoring and management, opinions, including opinions about the beliefs of the other individuals of the triad, and communication among the triads. Modifications of the interview guidelines after pre-test interviews were minor, only concerning the sequence of a few questions, and the rephrasing of some questions as open-ended questions.

### Interviewer training

NP is an experienced qualitative interviewer. KH and AL were trained by NP in an interviewer workshop where two patients were interviewed using the guideline for patient interviews developed in Bonn. KH and AL received feedback about their interviewing techniques from the respective other researchers after the pilot interviews.

### Data collection

Interviews were conducted between December 2014 and July 2015 by AL (Master of Public Health), KH, and NP (both postdoctoral psychologists). Patient interviews lasted between 24 and 121 minutes in Bonn, between 32 and 111 minutes in Hamburg, and between 16 and 54 minutes in Leipzig. Patients were interviewed face-to-face in the accommodations where they lived and the interviews were audiotaped.

### Data analysis

Audiotapes were transcribed verbatim by a research assistant following designated transcription rules. All transcripts were checked for accuracy and corrected where required (e.g., language corrections). They were read repeatedly and important contents of all interviews were summarised in an abstract to get an overview over each triad and to facilitate the identification of important topics. The unabbreviated transcripts were analysed using qualitative content analysis [16] to condense the large amount of data to identify the main themes. The coding was conducted using MaxQDA version 11 (Verbi GmbH). At first, deductive categories were developed based on the literature and based on plausible investigator assumptions about the research objectives (e.g., communication between GP and patient, positive effects of PIM on quality of life, and barriers to deprescribe PIM drug). Inductive categories were added during the coding process when relevant themes emerged, or sub-categories of higher level deductive categories were identified. Categories were described in code memos. Contextual factors were grouped into factors that might increase the probability of chronic intake of PIM, derived from PIM interviews, and factors that might decrease the probability of chronic intake of PIM, derived from non-PIM interviews. The development of deductive categories was initially conducted by KH and FJ in close collaboration with NP and AL. The patient interviews were content analysed at the study site in Bonn (by KH) in close collaboration with the study site in Hamburg (NP) and Leipzig (AL). Inductive categories were developed by KH in close collaboration with FJ during the transcript reviews. The results of the qualitative content analysis were discussed during two interprofessional meetings of the CIM-TRIAD study group at different study sites (by KH, NP, AL, MS, FJ, SRH, BH, and DP) to secure intersubjective comprehensibility and credibility.

### Ethical considerations

The study was approved by the local Ethics Committees (Ethics Committee of the Medical Faculty of the University of Bonn: July 14^th^ 2014, 169/14; Hamburg Medical Association: October 8^th^ 2014, MC-251/14; Faculty of Medicine, University of Leipzig: August 27^th^ 2014, 269-14-25082014). All participants gave a written, informed consent to participate in the study which included their explicit consent to contact and interview their GPs and associates.

## Results

Positive features of PIM, characteristics that maintain the intake of the medication, barriers to deprescribe PIM, external actors supporting PIM intake, and system-related factors reported by the patients were identified as main categories of contextual factors that might increase the probability for chronic use of PIM (see Table 3).

**Table 3:** Contextual factors that might increase the probability of chronic intake of PIM (d = deductive category, i = inductive category).

Positive features of PIM (d)	Maintaining characteristics of medication intake (d)	Barriers to deprescribe PIM (d)	External actors supporting PIM intake (d)	System-related factors (d)
PIM intake for many years (d)	Prescription of PIM on patient request (d)	PIM is not rated as problematic medication (i)	GP´s prescription of PIM due to patient request despite own reservation (d)	Acceptance of prescription of previous GP or medical specialist (d)
Positive effects of PIM (e.g., particular efficacy) (d)	Risk-benefit weighting of PIM intake (d)	Patient does not care about side effects of PIM (i)	GP rather unconcerned about PIM (i)	More permissive attitude towards PIM of antecedent physicians (i)
Positive side effects of PIM (i)	Low dose intake of PIM (i)	Alternative treatments are not utilised (d)	Long-term prescription of PIM without personal contact between patient and GP (i)	Some PIM are over-the-counter (OTC) products (i)
Good tolerance of PIM (d)	Intake of PIM only if required (i)	Resistance against cessation of PIM (d)	Private prescription vs. cost acquisition by health insurance (i)	
Positive effect of PIM on quality of life (d)	Intake of PIM or its indication unknown (lack of knowledge) (i)	Dependency or failed discontinuation of the medicine (d)	Ageism by the GP (d)	
		Ageism by the patient (i)	Relatives support intake of PIM (i)	

### Positive features of PIM

Positive features of the PIM perceived by the patients might contribute to the chronic use of that drug. For example, the perception of positive features might support the patient´s wish for a continuation of the prescription.

#### PIM intake for many years

Several patients reported a long duration of intake of their PIM. This might indicate that the patients tolerated the drug well.

“Well, but that was twenty, more than twenty years ago.” (P3, Bromazepam)

#### Positive effects of PIM

Several patients reported that they perceived a positive effect of their PIM (frequently, but not exclusively for benzodiazepines). Some patients reported that they perceived a distinguished efficacy and a strong or very prompt effect (also in comparison to other drugs), particularly for benzodiazepines and hypnotics.

“Cause this is an agent that truly helps, you, you can feel that. 15 minutes after I have taken that, I calm down, yes.” (P11, Bromazepam)

#### Positive side effects of PIM

A few patients reported positive drug side effects of their PIM besides the intended main effect of the drug. Besides the quotation below, positive side effects of benzodiazepines on cardiovascular symptoms, such as high blood pressure, were reported.

“And you sleep well with it, you know? […] This was an effect as well, because there were times I couldn´t sleep at all.” (P7, Trimipramin)

#### Good tolerance of PIM

Several patients reported that they tolerate their PIM very well. Some patients perceived a better compatibility of their PIM compared to an alternative medication.

“I went to a specialist years ago, a neurologist, you know […]. And he prescribed me pills I couldn´t tolerate. First because of my heart and they made me throw up. And then I said "I don´t like this stuff, I take [Bromazepam] ".” (P3, Bromazepam)

#### Positive effect of PIM on quality of life

Several patients, especially those who took benzodiazepines, but also hypnotics, reported positive effects on the quality of their lives.

“To ease my mind, to do me good (laughs).” (P1, Bromazepam)

### Maintaining characteristics of medication intake

There might be characteristics of the medication intake behaviour of patients that contribute to the maintenance of chronic prescription and of chronic use of PIM.

#### Prescription of PIM on patient request

Some patients reported that they request the prescription of their PIM with variation in the intensity of their appeals.

“I was demanding that. I said [to him] "My wife always got your prescription for that [drug]", and then I said "and she always fell asleep immediately".” (P4, Bromazepam)

#### Risk-benefit weighting of PIM intake

Especially patients who took benzodiazepines and hypnotics reported at least some awareness of potential risks due to the medication intake. They were weighing up these risks by also considering the benefits of the PIM.

“I don´t want to go over the top, too. That is, I don´t want to be hooked on that.” (P23, Doxylamin/ Zopiclon)

#### Low dose intake of PIM

Several patients that chronically used benzodiazepines reported a low dose intake of these drugs.

“I always had Bromazepam 6, thus 6 milligramme, and then lowered it to 3 milligramme on my own initiative. So that is a tiny dose only.” (P11, Bromazepam)

#### Intake of PIM only if required

Beside a low dose intake of PIM, “reasonable users” might also take the PIM only if it is required. Again, several patients that chronically used benzodiazepines reported that they take these drugs only as needed. The tendency to minimise one´s own drug usage should also be considered in this context.

“I sometimes don´t need it for one or two weeks, and (…), but when I know beforehand “you won´t fall asleep”, for whatever reason, then I take half or a whole according to circumstances.” (P1, Bromazepam)

#### Intake of PIM or its indication unknown (lack of knowledge)

Interviews occasionally revealed that patients were not informed about the intake of the PIM at all (some of these patients were living in a nursing home and were not in charge of their medication anymore) or they did not know the indication of their PIM (although they knew that they were taking it). A lack of knowledge might contribute to the chronic usage of PIM as patients are probably hindered to initiate its cessation.

“I´m taking that for years already, but (.) I don´t know at all for what/ (…) It is for your head somehow, but/.” (P19, Piracetam)

### Barriers to deprescribe PIM

We identified different barriers that might prevent the deprescription of the PIM.

#### PIM is not rated as problematic medication

This category represents the circumstance that the PIM has not a unique value or a special benefit for the patient so that cessation might be accomplished if its risks would be aware.

“And this other one, I know that, which is against my incontinence, if it does or does not help, I don´t really know. But I also do not have the feeling that it strains me, let´s put it this way.” (P18, Solifenacin)

#### Patient does not care about side effects of PIM

Several patients with chronic usage reported that they perceived side effects of their PIM. However, the intensity and valence of these side effects varied and side effects did not affect the patients in a sufficiently strong way to create a wish to stop the intake.

“I´m sometimes a little bit insecure then, you know? […] But I made arrangements therefore, I know exactly where my slippers are, […]. So, that´s working well.” (P2, Zopiclon)

“What I quite always have then, dry mouth, dry eyes, stuff like that, you know? That should also be caused by the medication, but I don´t certainly know.” (P7, Trimipramin)

#### Alternative treatments are not utilised

Some patients that chronically used PIM reported that they know medication-based and non-medication-based alternative treatments (e.g., autogenic training, herbal drugs, and psychotherapy), but that these alternative treatments either were not effective or they were used in addition to the intake of the PIM.

“Yes, I know autogenic training, stuff like that, (..) but, I know that. I learned that once, but I hardly make use of it yet.” (P1, Bromazepam)

#### Resistance against cessation of PIM

Several patients that chronically used PIM reported a hesitation or a resistance against the cessation of the medicine.

“They, they (.) in the hospital. They say, I would not be willing to stop taking these tablets. It´s mentioned in every report.” (P3, Bromazepam)

I: “I mean, what, what sort of argues against the cessation of it?” P: “When the depression returns, I´m afraid of that.” (P7, Trimipramin)

#### Dependency or failed discontinuation of the medicine

Several patients that chronically used benzodiazepines or hypnotics in particular reported some sort of dependency on the drug. A few patients reported that they tried to discontinue the intake of the PIM but that they failed to accomplish this.

“I´m always taking that. Well, I tried to do without it before, but that doesn´t work.” (P2, Zopiclon)

“I don´t know, if it is psychogenic or, but I do think so, that it is being addicted.” (P11, Bromazepam)

#### Ageism by the patient

Some patients made fatalistic statements that implied ageism as they reported that different medication-based efforts or alterations were not worthwhile due to their own age or due to already established impairments.

“And she doesn´t like doing that, because she just says "It damages your brain". What can you damage in mine anymore, I´m going to be ninety years old soon.” (P12, Bromazepam)

### External actors supporting PIM intake

Besides the patient, physicians and relatives or other associates of the patient might influence the medication of the patient.

#### GP´s prescription of PIM due to patient request despite own reservation

Some patients reported that their GPs expressed concerns about certain drugs, particularly in the context of benzodiazepines and hypnotics. These GPs prescribed the PIM reluctantly and only on patient request.

“[…] she prescribes them to me though, because she knows that I´m hooked on it, but I do not believe that she would initially prescribe it for someone, I do not believe that.” (P11, Bromazepam)

#### GP rather unconcerned about PIM

Some patients reported that their GPs seemed not to point out that the drug might potentially entail special risks or side effects, but instead were rather unconcerned about the dosage for example.

“He also send me, I mean doctor X, to a neurologist because of this [Trimipramin]. And he was listening to me and then he said "Well, if it is good for you, just take more of it". I thought then that I don´t have to visit a doctor for advice like that. […] Doctor X said "Just take, just take one whole tablet, if you want to".” (P7, Trimipramin)

#### Long-term prescription of PIM without personal contact between patient and GP

Some patients reported that they obtain prescriptions for their PIM without regular personal contact. For example, a rather uncomplicated request by phone was described for benzodiazepines and other PIM (i.e., Sotalol).

“I call them and it is prescribed (laughs). […] They know that I, the girls know that I don´t come around regularly because I can´t.” (P1, Bromazepam/Flurazepam)

#### Private prescription vs. cost acquisition by health insurance

Some patients received their (benzodiazepine) PIM as private prescription and needed to pay the costs by themselves, whereas others received a prescription for the same drug payed by their health insurance.

“I get it from the health insurance.” (P3, Bromazepam)

“The health insurance A also does not pay for that.” (P4, Bromazepam)

#### Ageism by the GP

Some patients reported that their age was used as an argument against PIM discontinuation or for continuation by their GPs.

“He prescribed it to me anyway and then he always said afterwards "Ah, do you know what? Shall we cancel that? No" he said, "we won´t do that. You are so old now, it doesn´t matter anymore. Just go on taking it".” (P7, Trimipramin)

#### Relatives support intake of PIM

A few patients reported that their relatives supported the intake of the PIM. Relatives either agreed that the intake of the PIM was indicated or provided patients with the drug in situations in which patients could not get it on their own (e.g., while staying in a hospital). Initial recommendations of relatives on the intake of PIM were also reported.

“I told my son that he should bring them along. […] It was over here. And then I took it as needed.” (P3, Bromazepam)

“My [relative x] is the only one, she says, well, at the age of [x] why should you plague yourself and wander around, if you are alright with a quarter or a half of it [Bromazepam].” (P11, Bromazepam)

### System-related factors

#### Acceptance of prescription by previous GP or medical specialist

Several patients reported that they were taking their PIM for several years and that the medication was initially prescribed by a previous GP or a medical specialist (e.g., cardiologist or neurologist).

“When I came to doctor Y (…), I already had that from doctor X.” (P7, Trimipramin)

#### More permissive attitude towards PIM of antecedent physicians

A few patients reported that their former physicians had a more permissive attitude towards PIM. Patients received their initial PIM prescription from these antecedent physicians and their present physicians continued this medication.

“My former physician was an elderly man and he loved to prescribe this [Bromazepam] and certainly I took it as well.” (P11, Bromazepam)

#### Some PIM are over-the-counter products

Some patients reported that they bought their PIM (Doxylamin) as over-the-counter (OTC) product in pharmacies. Some of these patients did not inform their GPs about the intake of this drug on purpose.

P: “It was prescribed by my previous physician and they are available without prescription. And I´m also taking them since then.” I: “But you did not talk about that with doctor X so far?” P: “No, no, I will beware of doing that.” (P11, Doxylamin)

Beside the above-mentioned contextual factors that might increase the probability of chronic intake of PIM, we also found characteristics reported by patients without PIM intake that might indicate contextual factors that possibly decrease the probability of (chronic) intake of PIM (see Table 4).

**Table 4:** Contextual factors that might decrease the probability of chronic intake of PIM (d = deductive category, i = inductive category).

Health-related behaviour (d)	Patient-GP-interaction (d)	Medication-related attitudes and knowledge (d)
Frequent medical examinations (i)	Agreement or consultation on dosage, side effects, alterations, and discontinuation of a drug (d)	Reservation against hypnotics, analgetics, and psychotropic drugs (i)
Own obligation to report to GP (i)	Mutual agreement of patient and GP on having a test phase for newly prescribed medication (i)	No PIM prescription despite agitation, sleep disturbance, or depression (i)
Critical attitude towards medication in general, but conscientious usage of necessary drugs (i)	Agreement upon self-medication and medical specialist prescriptions (d)	Awareness of side effects and interaction of medicines (d)
Adherence to GP´s instruction or non-adherence after consulting GP (d)		
Refusal of (pronounced) self-medication (d)		

### Health-related behaviour

#### Frequent medical examinations

Patients without (chronic) PIM usage more often reported that they attach great importance to frequent medical examinations.

“That doctor X used to do it regularly once a year and also do/, if I´m taking medicine. And then I think one should have a look once a year how the blood is getting along with it, you know?” (C1, non-PIM)

#### Own obligation to report to GP

Patients without (chronic) PIM usage more often mentioned their own obligation to report relevant information to their GP. In contrast, some patients with chronic PIM usage stated that they tried to bother their GPs as least as possible.

“I informed the general practitioner about all of it, so that he has an overview of medicines and side effects and stuff like that.” (C20, non-PIM)

#### Critical attitude towards medication in general, but conscientious usage of necessary drugs

In particular, patients without (chronic) PIM usage reported a critical view on medication and an ambition to take the least number of drugs as possible. However, several of these patients also reported that they took required drugs conscientiously and sometimes described certain groups of agents as important.

“Well, as I said, I am against pills, but I also accept their necessity in the end.” (C2, non-PIM)

#### Adherence to GP´s instruction or non-adherence after consulting GP

Patients without (chronic) PIM usage reported that they either adhered to the instructions of their GPs or reported non-adherence after consulting their GPs. As a counter-example, a patient that chronically used PIM continued to take Ibuprofen, although a physician explicitly warned her against the intake of this drug due to gastric burden during a hospital stay.

I: “And how did doctor X respond then when you sort of let´s say confessed it to her?” P: “She just quickly looked at me, but didn´t say anything further. Wasn´t complaining either or something, she said "Ok, but we won´t write discontinued but only break instead" [Allopurinol].” (C15, non-PIM)

#### Refusal of (pronounced) self-medication

As already mentioned above, patients without (chronic) PIM usage reported a critical attitude towards drugs. The refusal of self-medication might be related to that attitude and was reported by patients without (chronic) PIM usage repeatedly, although it was also expressed by some patients with chronic PIM usage.

“Just those stuff, that you are buying additionally. Yes, and I do not buy anything in the drugstore. What´s all this good for?” (C9, non-PIM)

### Patient-GP-interaction

#### Agreement or consultation on dosage, side effects, alterations, and discontinuation of a drug

Patients without (chronic) PIM usage reported agreements or consultation with their GPs on various occasions and at different stages of the medication process.

**Dosage**

“I could not get by with that at all. And then he tried out that one and with that one I´m fine. I had the twofold dosage first, but that was too much.” (C8, non-PIM)

**Side-effects**

“It´s the same with tablets for cholesterol, I can´t stand those at all. When I/, I had to/, take something against cholesterol. Well, I was really not fine then. And then he said "Stop it immediately, it [cholesterol] is also not too high".” (C11, non-PIM)

**Alterations**

“I said "Doctor, this great amount of pills". […] Sometimes, I took twelve pills a day. "Well", he said, "Miss X, I will now prescribe you something, where you have three tablets in one".” (C23, non-PIM)

**Discontinuation of a drug**

P: “I will ask him then, what it´s all about and that´s it. And then, well, "You have to take them indeed", yes, then I will do that.” I: “Ok.” P: “But also nothing more than that. And if it´s feasible somehow they will be removed as quickly as possible.” I: “On your own responsibility? Or //would you talk about that//?” P: “//No, then I would// talk about that with the general practitioner of course.” (C6, non-PIM)

#### Mutual agreement of patient and GP on having a test phase for newly prescribed medication

Particularly, patients without (chronic) PIM usage reported mutual agreements with their GP on trying out a new medication for a specified period of time and having another meeting afterwards to discuss and to decide whether the prescription of the drug should be continued.

“She explains to me how its action is supposed to be and how the new one is supposed to be tried out. And if it´s not doing good, I just ought to/ after two, three, four days, depending on what kind of drug it is, I have to/ she said "Terminate it, let me know".” (C19, non-PIM)

#### Agreement upon self-medication and medical specialist prescriptions

Some patients without (chronic) PIM usage reported that they inform their GPs about OTC drugs that they take or about medication that was prescribed by medical specialists. As a consequence, patients ensure that their GPs have a complete overview over their medication intake. As a counter-example, a patient that chronically used PIM did not inform the GP about recommencing the intake of Ibuprofen despite an already existing medication against pain.

“If someone prescribes something to me I call her [GP] "Pardon me, can I take that?".” (C3, non-PIM)

### Medication-related attitudes and knowledge

#### Reservation against hypnotics, analgetics, and psychotropic drugs

Several patients reported a sceptical view of using hypnotics, analgetics, and psychotropic drugs. As some of these drugs must be considered potentially inappropriate, patients who strictly avoid these drugs will at least not get a PIM prescription for sleep, pain, or psychotropic medication.

P: “That is (…) I do not take sleeping pills or stuff like that. I´m doing that on principle”. I: “Yes, on principle?” P: “Yes, on principle. Not any pain-killing tablets as well.” (C2, non-PIM)

#### No PIM prescription despite agitation, sleep disturbance, or depression

Although one might counter that those patients with a sceptical view on psychotropic drugs might be just those who do not need the respective medication, it has to be replied that, in fact, some of the patients who reported a critical view on psychotropic drugs did report past or present mental stress but decided on alternative treatment methods.

“I was afraid to get addicted to it. That is why I just didn´t take it anymore. If it´s so good/ so that I/ I was already retired then and I have enough work here, so that I could let off steam then.” (C15, non-PIM)

#### Awareness of side effects and interaction of medicines

Although patients with chronic usage of PIM were also aware of possible risks related to medicines, several patients without chronic usage of PIM explicitly reported a heightened awareness of side effects and possible drug interaction.

“All drugs do have side effects. I haven´t seen one that doesn´t yet. Their use should be as limited as possible.” (C8, non-PIM)

## Discussion

Contextual factors that might increase or reduce the probability of chronic PIM use were derived from interviews with patients with and without chronic PIM use.

### Factors that might increase chronic PIM use

Positive features of the PIM motivated the patients to continue its use. Positive main and side effects, a good tolerance, a long-lasting PIM intake, and positive effects of PIM on quality of life were sub-categories of this contextual main factor. In case of a long-lasting intake, PIM might have been prescribed initially in younger ages and might not have been potentially inappropriate then due to the patient´s age. Good tolerance of PIM might contribute to the continued prescription, but adverse effects might develop later on due to aging processes. However, a PIM prescription at a younger age might also produce habituation, and the awareness of adverse effects might be reduced over time. Positive effects of PIM on the quality of life were predominantly reported for benzodiazepines and hypnotics in our study. Cook et al. [17] found that many older patients with long-term benzodiazepine use attach great importance to the positive effects of benzodiazepines, while negative side effects are neglected. “Means to cope with stress or anxiety” or the “ability to make them feel more like themselves” were identified in this study ([17] p. 1096), which are similar to positive effects on quality of life reported in the present study. Hence, patients that chronically use benzodiazepine PIM might be “active users” due to the appreciated positive drug features, whereas patients that use other PIM might be less active or rather “passive users”. In another qualitative study by Cook et al. [18], physicians minimised concerns regarding potential problems of benzodiazepine use and justified long-term use in the elderly (also due to a lack of signs of addiction). The patients´ resistance against benzodiazepine discontinuation was anticipated by the physicians who also did not consider it a central focus of their work to monitor or restrict benzodiazepine prescription in elderly patients [18]. Mah and Upshur [19] found more discordant patient and physician perceptions of long-term benzodiazepine use as patients rated these drugs more positive. Although benzodiazepines are PIM due to the high risk of adverse drug effects, it should be mentioned that ceasing a long-lasting usage in old age can also bear risks and requires medical management (e.g., [20]).

The interviews of patients with chronic PIM use often suggested that the patients were taking their drugs in a reasonable way or that the patients had a lack of knowledge regarding the PIM. Reports of patients on low dose benzodiazepine intake might be due to an underestimation of the real intake or due to low dose dependency, but it might also be a mode of drug intake that maintains chronic use, as these patients might be “reasonable users”. It seems plausible that GPs might be induced to prescribe a potentially problematic drug for rather careful patients, whereas GPs might not prescribe the same drug to noncompliant patients. Patients who do not know that they are taking a PIM or do not know its indication, might not associate potential side effects with the PIM and might therefore not have the opportunity to speak up for its cessation. A lack of knowledge regarding medication might also be associated with insecurity in elderly patients as Modig et al. [8] identified “deficient information”, “distrust”, and “lack of availability” as sub-categories of “insecurity with information” associated with the potentially modifiable factors “too short consultations, discontinuity, lack of availability for questions or opportunity to contact the physician if adverse effects were suspected” (p. 9).

Barriers to deprescribe the PIM drug were also reported. Some patients did not care about the side effects of their PIM. Other patients rejected alternative treatments or resisted the PIM cessation (e.g., due to dependency). In a study by Leydon et al. [21] on selective serotonin reuptake inhibitors (SSRI), “patient uncertainty about benefits of and continued need for medication”, “barriers to stopping”, and “the importance of GP´s role in facilitating cessation” were identified as main topics. As also reported by patients in our study, the fear of relapse and the fear of withdrawal symptoms were also reported in this study [21]. Verbeek-Heida and Mathot [22] also found that the fear of stopping the intake of SSRI exceeded the fear of its continuation.

External actors supported the PIM use and contributed to it as well. Some patients reported that although their GPs prescribed them PIM, they informed the patient about adverse drug effects and expressed their concerns. Other GPs seemed rather unconcerned about PIM and some prescribed it without personal contact over longer periods of time. Private prescriptions and cost acquisition by health insurance were both reported by (benzodiazepine) patients. This might be a strategy to regulate the intake of a drug (eventually encouraging a lower drug dosage, if the patient has to pay for it) or to conceal the prescription of certain drugs (e.g., from health insurances). Ageism expressed by the GP was also identified in another study as a sub-category that underlies the inappropriate use of medicines [12]. Some patients in our study also expressed statements of ageism, which might interfere with PIM cessation as a fatalistic outlook on the need to change drug schedules due to the own high age was implied. In rare cases, relatives supported the use of PIM.

System-related factors contributed to the chronic PIM use in some cases. For example, PIM drugs that can be bought OTC were consumed by some patients without informing the GP because the patients expected their disapproval. If drugs such as Doxylamin would be only available on prescription, some patients would possibly dispense with it. The problem that some PIM drugs can be bought OTC in the United States of America was also discussed by other authors [23]. Mental disorder, female gender, and number of long-term medication were patient factors that increased the probability of PIM prescription in another German study [24].

### Factors that might decrease chronic PIM use

Patients without PIM use were also interviewed to generate hypotheses about “successful pathways” of the medication process. Characteristics of health-related behaviour, patient-GP-interaction, and medication-related attitudes and knowledge were identified as contextual factors. However, we want to point out that some of these characteristics were also expressed by patients with chronic PIM use. One potentially relevant health-related patient behaviour was a critical attitude towards medication in general combined with a conscientious usage of necessary drugs as these patients probably will not take dispensable medicine. Patients who adhere to GP instructions and give their GP a complete drug overview might reduce their risk for drug-drug-interactions. A patient-GP-interaction that is characterised by agreements on various aspects of medication might give patients the opportunity to report on adverse effects, but probably also requires a higher level of commitment of the patients. However, if practiced, agreements might reduce the risk of chronic PIM use, for example if (subtle) side or adverse drug effects exist.

Spinewine et al. concluded that “improvements targeted at the abilities of individuals, better doctor-patient and doctor-doctor relationships, and systems for transferring information between care settings” will lead to a more appropriate use of medication ([12] p. 1). These results are in good accordance with our results regarding “successful pathways” of drug prescribing as we also identified mutual agreements and close communication between patient and GP, which should stimulate treatment review, as possibly advantageous characteristics. Britten et al. [7] found that misunderstandings often occurred during the prescription of drugs and both patients and physicians contributed to this (i.e., “patient information unknown to doctor”, “doctor information unknown to patient”, “conflicting information given”, “disagreement about attribution of side effects”, “failure of communication about doctor´s decision”, “relationship factors”; p. 485). Although these results were not PIM-specific, it underlines the importance of close communication between patient and GP to prevent misunderstandings and to identify and eliminate side effects of the drugs.

Medication-related attitudes that were more frequently reported by patients without PIM use dealt with a personal reservation against hypnotics, analgetics, and psychotropic drugs, although a few patients without PIM use reported psychological stress for which alternative medical or non-medical treatments were used. Hence, although a documented mental disorder increased the risk for the prescription of PIM in another study [24], there are patients that favour alternative non-medical treatments for psychological distress or sleep disturbances. Former studies showed that psychotropic drugs such as psycholeptics/psychoanaleptics [4] and sedatives/hypnotics [24] account for a high percentage of PIM prescription. Hence, reservation against psychotropic drugs should reduce the possibility for PIM intake, at least regarding drugs of these agent groups.

### Strengths and limitations of the study

Audioconferences, an interviewer training and personal meetings provided extensive and interdisciplinary exchange and confirmability during the development and implementation of the interview guidelines. The fact that modifications of the interview guidelines after pre-test interviews were mostly not required was interpreted as an indicator of credibility. The content analysis was conducted only by one coder for the patient perspective. Although this was a clear limitation of our study, we accomplished validity and confirmability of the coding by ensuring an intensive exchange on the coding system, the results and the presentation of the results during several audioconferences and personal meetings. Repeated reading and summarising of the full interviews also increased the confirmability of our results. Different PIM agents were included in our study and the duration of chronical PIM intake varied to some extent which should enhance the transferability of our results. Additionally, most of our participants were female which is in accordance with other studies showing a higher prevalence of PIM in women [4, 24]. Due to the design of our study, we did not include participants without associates and participants with severe cognitive impairment. Although these restrictions were necessary, they might have reduced the transferability of our results. The interview of triads contributed to the dependability of our results as cross-validation of reported contents automatically occurred to some extent. However, similarities between the group of PIM users and non-users, the dosage-dependent definition of some PIM, and the short- or long-term use of PIM, not regularly but as needed, might have diminished the dependability of our results.

The consideration of chronic PIM use enabled us to identify relatively stable contextual factors, instead of factors that might lead to short-term PIM use. We conducted a considerable number of patient interviews in our multi-perspective and multi-center qualitative study. The interview of triads and the inclusion of questions regarding quality of life were highly innovative. The integration of different professional perspectives on the obtained data was ensured by the different theoretical and educational backgrounds at the three study sites. The differing backgrounds of the patients lead to an enriched sample containing several pathways to chronic PIM use, or “successful” pathways without PIM use. As triads and different agent groups of PIM drugs were included, we planned a high number of interviews in advance. Therefore, saturation was not the only criterion for the number of interviews, but it was in good accordance with the rather high planned and presented number of participants. As there was an overlap to some extent between patients with chronic PIM use and those without, we aimed to find a reasonable compromise between higher-level conclusions and the potential danger of an oversimplification of the complex situation in real life contexts of the medication process in elderly patients. Although a lot of PIM cases were due to benzodiazepines, there was a wide range of different prescribed PIM agents in our sample overall.

### Conclusion

The results of our study should be relevant for both physicians and developers of guidelines and educational programs. The avoidance of initial and long-lasting PIM use by restrictive prescription and the sensitisation of the patient for the risks of these drugs might be indicated (e.g., specific side effects or drug-drug interactions). It might also be helpful to inform patients more actively about adverse effects of prescribed PIM and to inquire adverse drug effects routinely. Test phases for newly prescribed drugs and a routine follow-up appointment to discuss potential side effects should be arranged. A frequent drug treatment review and close communication between GPs and patients, including mutual agreements on prescribed drugs, should be pursued. Advantages of alternative treatment approaches should be emphasised by GPs. Concerns regarding the cessation of PIM might be addressed and modified in GP consultations. Discontinuation and withdrawal in case of dependency might be supported and facilitated by GPs. Ageism might be identified and reduced in educational programs and GPs might assist patients and relatives in the modification of drug-related ageism. In addition to improved specific drug education, strengthening global health literacy in patients as a more complex approach to empower patients might also be promising to reduce chronic PIM use. Our findings also might be useful to generate hypotheses about chronic PIM use which could be tested statistically in quantitative study designs.
